# Impairment of pulmonary vascular reserve and right ventricular systolic reserve in pulmonary arterial hypertension

**DOI:** 10.1186/1471-2466-14-69

**Published:** 2014-04-24

**Authors:** Enric Domingo, Juan C Grignola, Rio Aguilar, Christian Arredondo, Nadia Bouteldja, Manuel López Messeguer, Antonio Roman

**Affiliations:** 1 Area del Cor, Hospital Universitari Vall d’Hebron, Barcelona, Spain; 2 Physiology Department, School of Medicine, Universitat Autonoma, Barcelona, Spain; 3 Pathophysiology Department, School of Medicine, Hospital de Clínicas, Universidad de la República, Avda Italia 2870, PC 11600 Montevideo, Uruguay; 4 Department of Cardiology, Hospital de la Princesa, Madrid, Spain; 5 Department of Neumology, Hospital Vall d’Hebron, Barcelona, Spain

**Keywords:** Pulmonary hypertension, Dobutamine, Cardiovascular reserve, IVUS, Echocardiography

## Abstract

**Background:**

Exercise capacity is impaired in pulmonary arterial hypertension (PAH). We hypothesized that cardiovascular reserve abnormalities would be associated with impaired hemodynamic response to pharmacological stress and worse outcome in PAH.

**Methods:**

Eighteen PAH patients (p) group 1 NYHA class II/III and ten controls underwent simultaneous right cardiac catheterization and intravascular ultrasound at rest and during low dose-dobutamine (10 mcg/kg/min) with trendelenburg (DST). We estimated cardiac output (CO), pulmonary vascular resistance (PVR) and capacitance (PC), and PA elastic modulus (EM). We concomitantly measured tricuspid annular plane systolic excursion (TAPSE), RV myocardial peak systolic velocity (Sm) and isovolumic myocardial acceleration (IVA) in PAH patients. Based on the rounded mean + 2 SD of the increase in mPAP in our healthy control group during DST (2.8 + 1.8 mm Hg), PAH p were divided into two groups according to mean PA pressure (mPAP) response during DST, 1: ΔmPAP > 5 mm Hg and 2: ΔmPAP ≤ 5 mm Hg. Cardiovascular reserve was estimated as the change (delta, Δ) during DST compared with rest, including ΔmPAP with respect to ΔCO (ΔmPAP/ΔCO). All patients were prospectively followed up for 2 years.

**Results:**

PAH p showed significant lower heart rate and CO increase than controls during DST, with a significant mPAP and pulse PAP increase and higher ΔmPAP/ΔCO (p < 0.05). Neither hemodynamic, IVUS and echocardiographic data were different between both PAH groups at rest. In group 1, DST caused a higher ΔEM, ΔmPAP/ΔCO, ΔPVR, and ΔTAPSE than group 2, with a lower IVA increase and a negative ΔSV (p < 0.05). TAPSE correlated with mPAP and RVP (p < 0.05) and, IVA and Sm correlated with CO (p < 0.05). ΔEM correlated with ΔmPAP and ΔIVA with ΔCO (p < 0.05). There were two deaths/pulmonary transplantations in group 1 and one death in group 2 during the follow-up (p > 0.05).

**Conclusions:**

Pulmonary vascular reserve and RV systolic reserve are significantly impaired in patients with PAH. The lower recruitable cardiovascular reserve is significantly related to a worse hemodynamic response to DST and it could be associated with a poor clinical outcome.

## Background

Normal pulmonary circulation is characterized by low pressure and low vascular resistance. Mean pulmonary arterial pressure (mPAP) at rest is virtually independent of age and rarely exceeds 20 mm Hg (14 ± 3.3 mm Hg). In healthy individuals, passive distension of compliant pulmonary circulation and active flow-mediated vasodilation allows the pulmonary vasculature to accommodate increased cardiac output (CO) with only a modest increase in mPAP and a fall in pulmonary vascular resistance (PVR) [1-3]. Invasive hemodynamic monitoring during incremental exercise testing is technically difficult to perform and not routinely incorporated into clinical exercise testing. A recent systematic review has reported an age-dependent increase of mPAP that may exceed 30 mm Hg particularly in subjects aged ≥ 50 years, making it difficult to clearly define normal mPAP values during exercise [2]. In idiopathic pulmonary arterial hypertension (PAH), exercise capacity is markedly impaired due to an inefficient lung gas exchange (ventilation/perfusion mismatching with an increased dead space ventilation) and the inability of the heart to adequately increase pulmonary blood flow during exercise [4]. The pathophysiological mechanisms leading to an abnormal exercise response include an intrinsic abnormality in the pulmonary vasculature due to the pulmonary arterial (PA) wall remodeling [5] and a reduction in stroke volume and right ventricular (RV) ejection fraction [6]. Laskey et al. have demonstrated that both steady and pulsatile components of the PA vascular hydraulic load have considerable impact on exercise response in primary pulmonary hypertension [7]. It has already been reported that improvement in exercise tolerance in PAH patients with chronic therapy is independently related to improvements in pulmonary hemodynamics measured in exercise but not in resting conditions, suggesting an improve in the vascular reserve [8]. Lau et al. did not observe any significant beneficial effects of bosentan on arterial stiffness following 6-months of therapy [9].

Exercise echo-Doppler is being used with increased frequency in the assessment of patients with known or suspected pulmonary vascular disease, focusing on the change in Doppler-estimated PAP with exercise. However, there are surprisingly few data about RV function at exercise, especially considering that the impaired RV functional reserve could get involved in the mechanism of exercise limitation in PAH and other forms of pulmonary vascular diseases [10]. Recently, Blumberg et al. showed that the ability to increase the cardiac index during exercise is an important determinant of exercise capacity and it is linked to survival in patients with PH [11].

We hypothesized that abnormalities in cardiovascular reserve would be associated with impaired hemodynamic response to pharmacological stress and worse outcome in PAH. Therefore, the first aim of the present study was to perform RV systolic function assessment (echocardiography) and hemodynamic monitoring (right heart catheterization) including local elastic properties of proximal PA wall (intravascular ultrasound, IVUS) during dobutamine stress in patients with PAH. The second aim was to evaluate the association between the cardiovascular reserve and the outcome during two years follow-up.

## Methods

### Ethics statement

The investigation conforms with the principles outlined in the Declaration of Helsinki. The study protocol was approved by the Institutional Ethics Committee of the Hospital Universitari Vall d’Hebron (Barcelona), and all patients gave written informed consent.

### Study population

Eighteen consecutive patients with PAH (Dana Point group 1) under specific drug therapy who underwent a follow-up cardiac catheterization at our institution were included in the study from January 2007 to September 2009. The patients were in NYHA function class II-III, with no clinical and pharmacological changes in the last 4–6 months. Exclusion criteria were: refusal to participate in the study or being in NYHA function class IV. The diagnosis of PAH was made according to the standard algorithm including a right heart catheterization [12]. Causes of PAH were idiopathic PH (n = 12), PH related to connective tissue disease (n = 3), surgically corrected congenital heart disease (n = 1), PH associated with human immunodeficiency virus (n = 1) and porto-pulmonary hypertension (n = 1). Chronic medication included oral anticoagulants, diuretics on demand, bosentan, sildenafil, inhaled iloprost and epoprostenol, as well as combination therapies on clinical judgement. Age and sex matched control subjects were recruited initially referred for cardiac catheterization due to clinically suspected PAH, without any other heart or lung disease. They underwent the same invasive study protocol after documentation of normal pulmonary arterial hemodynamics.

All subjects underwent a routine right heart catheterization and simultaneous inferior lobe medium-sized elastic pulmonary artery IVUS in the supine position and breathing room air. A transthoracic echocardiographic study was performed concomitantly in PAH patients by a single experienced examiner. All variables were obtained at rest and during dobutamine stress test with simultaneous Trendelenburg (DST). DST consisted of low-dose dobutamine infusion (10 mcg/kg/min) in order to increase myocardial contractility and heart rate, and 30° Trendelenburg position in order to increase venous return (preload), both during 10 minutes, unless symptoms (shortness of breath, chest pain, systemic hypertension with systolic blood pressure ≥ 180 mm Hg or tachyarrhythmia other than sinus tachycardia) were observed. These three variables (venous return or preload, heart rate and myocardial contractility) are the leading factors responsible for the increase in CO during exercise [1]. We choose a low-dose dobutamine stress test with simultaneous Trendelenburg as an easy maneuver to induce a purely passive increase of pulmonary flow and a change in cardiac contractility, in order to assess the cardiovascular reserve [2].

PAH patients were prospectively followed up for 2 years. Physicians who carried out the clinical follow-up were blinded to the hemodynamic, IVUS and echocardiographic results.

### Hemodynamic and IVUS studies

A 7 F Swan-Ganz catheter (Edwards Lifesciences, USA) was inserted into a brachial vein and a 5 F end-hole catheter was inserted into the right radial artery to monitor systemic arterial pressure. Both catheters were connected to fluid-filled transducers, which were positioned at the anterior axillary line level and zeroed at atmospheric pressure. Right atrial, PAP and pulmonary capillary wedge pressures were all measured at end-expiration. CO was calculated using the Fick method. In patients with tricuspid regurgitation and low CO, such as those with PAH, the thermodilution method has not been reported to be more accurate than the Fick method [13], however in this series no patient presented tricuspid regurgitation greater than mild in the echocardiographic assessment nor at rest neither at peak stress. PVR was calculated as: (mPAP-pulmonary capillary wedge pressure)/CO and total pulmonary resistance (TPR) as: mPAP/CO. Pulmonary vascular capacitance (PC) was estimated by the stroke volume/pulse pressure ratio (SV/pPAP) [14]. The changes in mPAP were normalized by the changes in CO (ΔmPAP/ΔCO) during pharmacological stress, in order to interpret exercise-induced increases in mPAP relative to increases in blood flow [15].

IVUS examination was performed with an Eagle Eye Gold catheter 20 MHz, 3.5 F (Volcano Corporation, USA) with an axial resolution of 200 μm and an automatic pullback of 0.5 mm/s. The images were obtained from a segmental PA of the inferior lobe (elastic PA between 2–3 mm) [16-18] and stored in digital form. Both diastolic and systolic cross-sectional areas of the studied segment were analyzed off-line by two observers unaware of clinical and hemodynamic findings. We estimated IVUS pulsatility (IVUSp) as: (systolic-diastolic lumen area)/diastolic lumen area × 100. The physiological adaptation of the vessel wall to stress was estimated by the elastic modulus (EM) or pressure/elastic strain index (pulse pressure/IVUSp), an expression of the intrinsic PA wall viscoelastic properties and buffering function. Intra- and inter-observer validation of IVUS measurements in our laboratory has been previously published [16,18].

### Transthoracic echocardiography-Doppler study

Baseline and stress echocardiography was performed using commercially available equipment (Vivid 7 digital GE Medical System) with a standard 2D broad-band phased array M4S transducer and tissue Doppler imaging software. The transducer was maximally aligned to optimize endocardial visualization and spectral displays of Doppler profiles. Real-time 2-D and colour Doppler myocardial imaging were performed in the apical 4-chamber view as well as the parasternal short-axis and subcostal views. The predominantly longitudinal contractile pattern of the RV can be exploited to assess RV systolic function [19]. We estimated the global RV systolic longitudinal function by the tricuspid annular plane systolic excursion (TAPSE) measured from the systolic displacement of the RV free wall-tricuspid annular plane junction in the apical 4-chamber view M-mode recordings. Myocardial peak velocity during ejection phase (Sm) was assessed by tissue Doppler imaging in the basal segment of the RV free wall using spectral pulsed wave tissue Doppler recorded at a sweep speed of 100–150 mm/s. Myocardial acceleration during isovolumic contraction (IVA) was calculated as the maximal isovolumetric myocardial velocity divided by the time to peak systolic velocity, as previously described by Vogel et al. [20]. This method seems to be less load-dependent compared to the other two indices. Patients were required to hold their breath and images were obtained immediately after expiration for better image quality. All patients were in sinus rhythm, and an average of 3 to 5 measurements from consecutive cardiac cycles were employed for data analysis. All examinations were recorded digitally for subsequent blinded off-line analysis on EchoPAC GE Medical System. The estimation of intraobserver and interobserver reproducibility was analyzed.

### Cardiovascular reserve analysis

Cardiovascular reserve was expressed as the change (increase or decrease) in heart rate (ΔHR, chronotropic reserve), RV systolic function (ΔIVA, systolic reserve) and pulmonary vascular function (ΔEM and ΔPVR, vascular reserve) during DST when compared with rest [21].

### Statistical analysis

Continuous variables are expressed as mean ± SEM. Based on the rounded mean + 2SD of the increase in mPAP in our healthy control group during DST (2.8 + 1.8 mm Hg), PAH patients were divided, prior to analysis, into two groups according to their hemodynamic response to pharmacological stress: group 1 included those patients whose mPAP during stress increased > 5 mm Hg with respect to the resting value, and group 2 comprised those patients with a mPAP increase ≤ 5 mm Hg. Independent sample t-tests were used to compare differences between the control and PAH groups; and paired t-tests were used to compare the effects of stress maneuver within each group. Chi-squared was used for comparing proportions of patients. Intergroup variation was analyzed using one-way ANOVA.

The association between hemodynamic response (ΔCO, ΔmPAP) and cardiovascular reserve (ΔIVA, ΔEM, ΔPVR) were explored using linear regression analysis (Pearson coefficient). A two-sided *P* value < 0.05 was regarded as significant. Data analysis were carried out using SPSS 17.0 for Windows 7 software.

## Results

### Comparison between PAH patients and control subjects at rest and during pharmacological and positional stress

The age and gender of PAH subjects and control subjects were well matched (Table 1). Table 2 shows hemodynamic and IVUS data of both, control subjects and PAH patients at rest and during DST. PAH patients showed higher mPAP, pPAP, PVR, TPR and EM and lower PC and IVUSp than control subjects (*P* < 0.05). There were no significant difference in heart rate, SV, CO, right atrial pressure and pulmonary capillary wedge pressure between both groups.

**Table 1:** Demographic, anthropometric and clinical data of control subjects and patients with PAH

	**All PAH**	**PAH 1**	**PAH 2**	**Controls**
	**(n = 18)**	**(ΔmPAP > 5) (n = 9)**	**(ΔmPAP ≤ 5) (n = 9)**	**(n = 10)**
Demographic				
Age, years	51 ± 3.7	47 ± 5	55 ± 5	51 ± 1.8
Gender, M/F	7/11	3/6	4/5	4/6
BSA, m^2^	1.76 ± 0.05	1.73 ± 0.06	1.80 ± 0.05	1.80 ± 0.04
Functional status				
NYHA class II/III	10/8	5/4	5/4	
6MWD, m	390 ± 26	396 ± 41	383 ± 33	

BSA: body surface area; 6MWD: six minute walking distance.

**Table 2:** Hemodynamic and IVUS data of control subjects and patients with PAH at rest and during stress maneuver

	**PAH patients (n = 18)**		**Control patients (n = 10)**	
	**Rest**	**Stress**	**Rest**	**Stress**
CO, L/min	4.0 ± 0.3	5.6 ± 0.4^§^	4.7 ± 0.1	8.6 ± 0.2*^§^
HR, bpm	76 ± 3	104 ± 3.6^§^	73 ± 1.3	114 ± 1.5*^§^
SV, mL	55 ± 5.1	55 ± 2.2	64.5 ± 1.4	78 ± 2*^§^
mPAP, mm Hg	52 ± 4	61 ± 5§	15 ± 2*	18 ± 1.2*^§^
pPAP, mm Hg	49 ± 21	65 ± 6§	11 ± 3*	15 ± 1.1*^§^
PCWP, mm Hg	10.3 ± 1.0	7.3 ± 0.9	8.8 ± 0.6	11 ± 0.7
RAP, mm Hg	7.4 ± 0.9	7.7 ± 0.9	5.0 ± 1.2	6.5 ± 1.1
PVR, Wood units	12 ± 1.7	11.4 ± 1.9	3 ± 0.3*	1.9 ± 0.17*^§^
PC, mL/mm Hg	1.5 ± 0.26	1.1 ± 0.2^§^	6.2 ± 0.4*	5.5 ± 0.6*
TPR, Wood units	14.8 ± 1.9	13.1 ± 2.0^§^	3.3 ± 0.25	2.1 ± 0.16*^§^
IVUSp, %	33 ± 4.8	27 ± 4	52 ± 2.5*	85 ± 3.4*^§^
EM, mm Hg	184 ± 25	275 ± 36^§^	21 ± 1.9*	18 ± 1.7*^§^

*p < 0.05 between both groups in the same condition; ^§^p < 0.05 between both conditions in the same group.

CO: cardiac output; PC: pulmonary capacitance index; EM: elastic modulus; HR: heart rate; IVUSp: pulmonary arterial pulsatility; mPAP and pPAP: mean and pulse arterial pulmonary pressures; PCWP: pulmonary capillary wedge pressure; PVR: pulmonary vascular resistance; RAP: right atrial pressure; SV: stroke volume; TPR: total pulmonary resistance.

During DST healthy controls showed an increase of CO, SV and heart rate (*P* < 0.05) with a significant reduction in PVR and TPR, and improvement of IVUSp and EM (*P* < 0.05) (Table 2). These changes led to an attenuated increase in mPAP and pPAP. Mean systolic aortic pressure was 127 mm Hg at rest and 165 mm Hg during stress (*P* < 0.05). None of control subjects exceeded 20 mm Hg of mPAP at rest and 30 mm Hg during stress.

**Figure 1:**
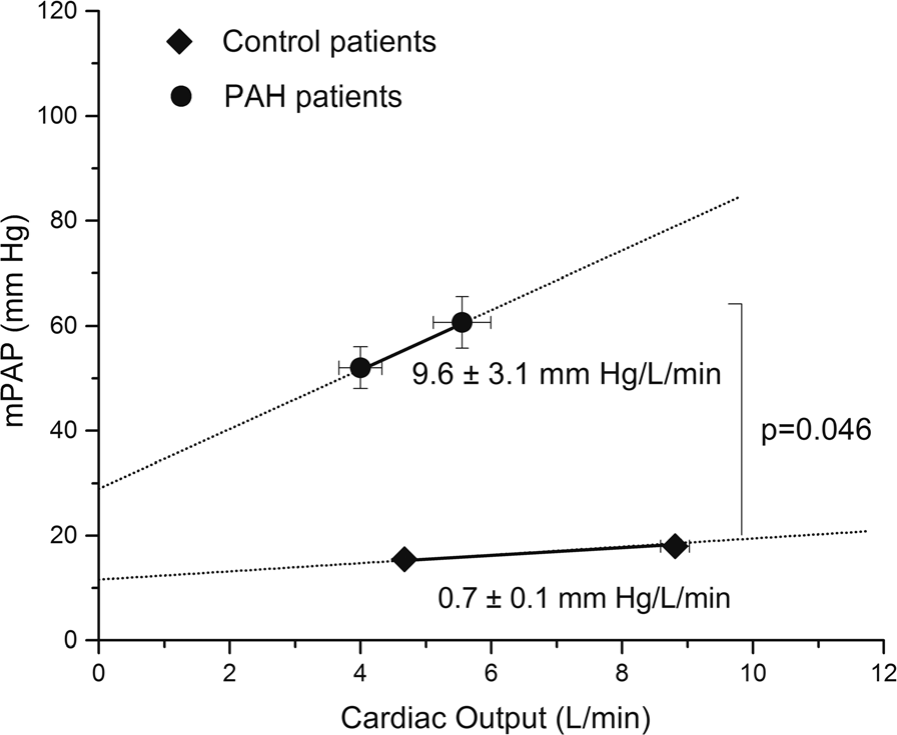
**Relationship between mean pulmonary artery pressure (mPAP) and cardiac output at rest and during pharmacological and positional stress.** (Filled circle: PAH patients; filled square: control patients).

All PAH patients tolerated the stress protocol. No dobutamine infusion had to be interrupted at the doses employed for this study and no complications were observed. No patients included in this study presented greater than mild tricuspid regurgitation (≤ grade 2/4), and there were no relevant changes in its severity during the complete protocol. Only six of 18 PAH patients increased SV, and the heart rate increase was significantly lower than control subjects (29 ± 3.8 vs. 41 ± 2 bpm, *P* = 0.034), therefore the CO increment was mainly dependent on heart rate increase. mPAP, pPAP and EM increased significantly and PC significantly decreased during stress (Table 2). However, they showed an increase in all the RV systolic function indexes during stress (TAPSE 16.8 ± 1.3 vs. 20.2 ± 1.1 mm, *P* = 0.02; Sm 12.0 ± 0.5 vs. 14.7 ± 0.8 cm ⋅ s^-1^, *P* = 0.003; IVA 3.8 ± 0.3 vs. 7.8 ± 0.9 m ⋅ s^-2^, *P* = 0.0003). The CO increase was less marked than in healthy controls (*P* < 0.05), and consequently, ΔmPAP/ΔCO was higher (9.6 ± 3.1 vs. 0.7 ± 0.1 mm Hg/L/min, *P* = 0.046) in PAH patients than in controls (Figure 1). High quality RV velocity curves were obtained both at rest and stress in 16 out of 18 patients.

### Changes in hemodynamic, IVUS and echocardiographic data in PAH patients according to ΔmPAP during pharmacological and positional stress

Nine patients increased mPAP > 5 mm Hg (group 1) and nine patients changed mPAP ≤ 5 mm Hg (group 2) during stress. Etiology of group 1 was 5 IPAH, 2 scleroderma-associated PAH, 1 congenital cardiac shunt and 1 HIV PH. Etiology of group 2 was 7 IPAH, 1 scleroderma-associated PAH and 1 porto-pulmonary hypertension. Neither demographic nor clinical differences between PAH group 1 and PAH group 2 were found (Table 1). Accordingly, neither hemodynamic nor IVUS data showed differences between both PAH patients groups at rest (Table 3).

**Table 3:** Hemodynamic, IVUS and echocardiographic data at rest and during stress maneuver of both PAH groups

	**PAH 1**		**PAH 2**	
	**ΔmPAP > 5 (n = 9)**		**ΔmPAP ≤ 5 (n = 9)**	
	**Rest**	**Stress**	**Rest**	**Stress**
CO, L/min	3.8 ± 0.5	5.0 ± 0.7^§^	4.2 ± 0.4	6.1 ± 0.5^§^
HR, bpm	76 ± 4	104 ± 5.3^§^	75 ± 4.6	105 ± 5.1^§^
SV, mL	54 ± 9	50 ± 9^§^	56 ± 6	60 ± 5
mPAP, mm Hg	54 ± 6.7	71 ± 7.2^§^	50 ± 4.7	51 ± 4.6*
pPAP, mm Hg	52 ± 6.7	76 ± 9.2^§^	45 ± 7.6	53 ± 6.4*
PCWP, mm Hg	10.6 ± 1.1	9.4 ± 1.1	10.3 ± 1.9	6.0 ± 1.3
RAP, mm Hg	7.9 ± 1.0	8.4 ± 0.8	6.9 ± 1.6	7.1 ± 1.2
PVR, Wood units	13.7 ± 2.8	14.9 ± 3.1	10.3 ± 1.7	7.9 ± 1.3*^§^
PC, mL/mm Hg	1.38 ± 0.4	0.9 ± 0.4^§^	1.6 ± 0.38	1.3 ± 0.2
TPR, Wood units	16.6 ± 3.1	17.0 ± 3.1	13.1 ± 2.2	9.2 ± 1.8*^§^
IVUSp, %	29 ± 5.7	26 ± 6.6	38 ± 7.8	28 ± 4.8
EM, mm Hg	211 ± 32	362 ± 55^§^	158 ± 39	187 ± 23*
TAPSE, mm	16.4 ± 1.9	20.8 ± 1.9^§^	20.3 ± 1.4	19.6 ± 1.1
Sm, cm ⋅ s^-1^	12.1 ± 0.64	15.3 ± 1.2^§^	12.8 ± 1.0	13.3 ± 1.0
IVA, m ⋅ s^-2^	3.3 ± 0.35	5.9 ± 0.7^§^	4.3 ± 0.5	9.9 ± 1.5*^§^

*p < 0.05 between both groups in the same condition; ^§^p < 0.05 between both conditions in the same group.

CO: cardiac output; PC: pulmonary capacitance index; EM: elastic modulus; HR: heart rate; IVA: myocardial isovolumic acceleration; IVUSp: pulmonary arterial pulsatility; PCWP: pulmonary capillary wedge pressure; mPAP and pPAP: mean and pulse arterial pulmonary pressures; PVR: pulmonary vascular resistance; Sm: myocardial peak systolic velocity; RAP: right atrial pressure; SV: stroke volume; TAPSE: tricuspid annular plane systolic excursion; TPR: total pulmonary resistance.

Both PAH groups increased CO during DST (*P* < 0.05), although without significant differences between them (1.14 ± 0.3 vs. 1.9 ± 0.3 L/min, NS). Only PAH group 1 showed a significant increase in mPAP, decreasing PC and increasing EM significantly, with no change in PVR and TPR. PAH group 2 decreased PVR and TPR without significant change in PC, IVUSp and EM (Table 3). PVR decreased in 2/9 patients in group 1 and in 8/9 in group 2 (*P* < 0.05). PC decreased in 9/9 patients in group 1 and in only 4/9 in group 2 (*P* = 0.08). Starting from a similar EM at rest, the EM of PAH group 1 was significantly higher than PAH group 2 (362 ± 55 vs. 187 ± 23 mm Hg, *P* < 0.05, Table 3) during DST.

RV systolic function indexes were similar between both PAH patients groups at rest. DST unmasked a significant lower increase of IVA of PAH group 1 with respect to PAH group 2 (5.9 ± 0.7 vs. 9.9 ± 1.5 m ⋅ s^-2^) (Table 3). Concomitantly, SV decreased (*P* < 0.05) in PAH group 1 during stress.

### Cardiovascular reserve responses during pharmacological and positional stress

Control subjects showed a higher chronotropic (Δheart rate) and systolic reserve (measured by ΔSV) than PAH patients (*P* < 0.05). The negative change in ΔEM and ΔPVR during stress revealed an increased vascular reserve associated with a low ΔmPAP/ΔCO ratio (Table 4).

**Table 4:** Cardiovascular reserve response of control subjects and both PAH groups

	**PAH 1**	**PAH 2**	**Control**
	**ΔmPAP > 5 (n = 9)**	**ΔmPAP ≤ 5 (n = 9)**	**(n = 10)**
ΔCO, L/min	1.1 ± 0.3	1.9 ± 0.3	4.2 ± 0.3*^§^
ΔHR, bpm	30 ± 5.2	28 ± 5.8	41 ± 2.1*^§^
ΔSV, mL	-4.1 ± 1.3	3.2 ± 2.8^†^	13.2 ± 2.8*^§^
ΔmPAP, mm Hg	17 ± 3.2	0.8 ± 0.9^†^	2.8 ± 0.3*
ΔpPAP, mm Hg	25 ± 4.8	7.2 ± 3.7^†^	4.0 ± 0.6*
ΔPVR, Wood units	1.2 ± 0.6	-2.4 ± 0.6^†^	-1.0 ± 0.2*^§^
ΔPC, mL/mm Hg	0.46 ± 0.11	0.3 ± 0.2	-0.7 ± 0.4
ΔTPR, Wood units	0.5 ± 0.48	-3.9 ± 0.7^†^	-1.2 ± 0.12*^§^
ΔIVUSp, %	2.8 ± 2.2	9.4 ± 6.7	32.5 ± 3.2*^§^
ΔEM, mm Hg	151 ± 29	30 ± 36^†^	-3 ± 1.2*
ΔmPAP/ΔCO, mm Hg/L/min	19.5 ± 3.9	0.3 ± 1.3^†^	0.7 ± 0.07*
ΔTAPSE, mm	4.4 ± 0.9	0.09 ± 1.1^†^	
ΔSm, cm ⋅ s^-1^	3.4 ± 1.2	1.8 ± 0.8	
ΔIVA, m ⋅ s^-2^	3.2 ± 0.9	4.9 ± 1.4	

^†^p < 0.05, PAH 1 vs. PAH 2; *p < 0.05, Control vs. PAH 1; ^§^p < 0.05, Control vs. PAH 2.

CO: cardiac output; PC: pulmonary capacitance index; EM: elastic modulus; HR: heart rate; IVA: myocardial isovolumic acceleration; IVUSp: pulmonary arterial pulsatility; mPAP and pPAP: mean and pulse arterial pulmonary pressures; PVR: pulmonary vascular resistance; Sm: myocardial peak systolic velocity; SV: stroke volume; TAPSE: tricuspid annular plane systolic excursion; TPR: total pulmonary resistance. Δ = delta.

Considering all PAH patients, resting EM, but neither PVR nor PC, was correlated with ΔmPAP (r = 0.49, *P* < 0.005) and ΔCO (r = -0.72, *P* < 0.0001).

Global cardiovascular reserve was impaired in PAH group 1, showing the higher increase in ΔEM, higher ΔmPAP/ΔCO ratio, with a negative change in ΔSV and a positive change in ΔPVR and ΔTPR. By contrast PAH group 2, showed some extent of cardiovascular reserve, illustrated by the changes of ΔEM, ΔPVR, ΔmPAP/ΔCO ratio and ΔSV with respect to control patients (Table 4).

**Figure 2:**
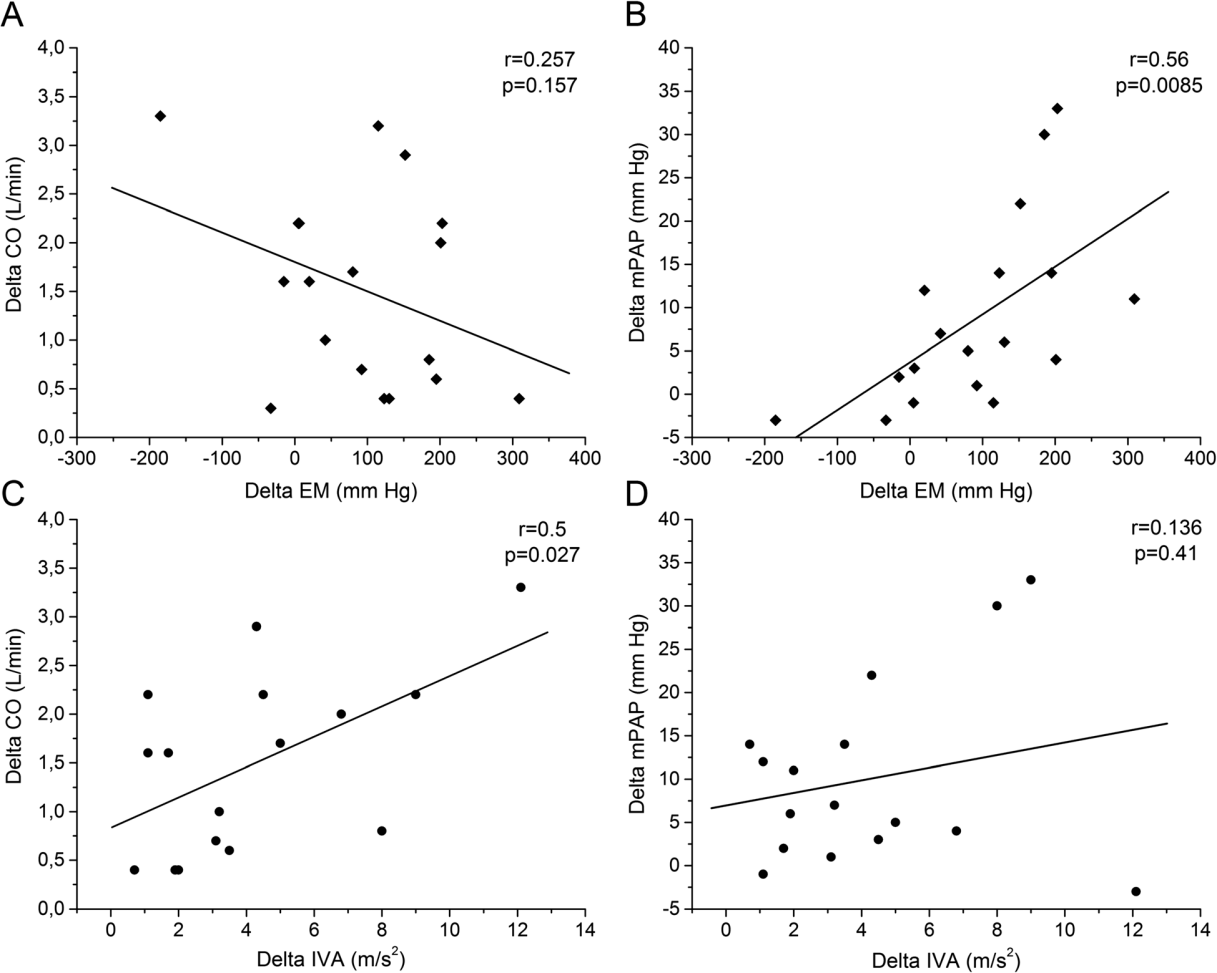
**Relationship between hemodynamic response and cardiovascular reserve. A**. Correlation between delta elastic modulus (ΔEM) and delta mean pulmonary artery pressure (ΔmPAP); **B**. correlation between ΔEM and delta cardiac output (ΔCO); **C**. correlation between delta myocardial isovolumic acceleration (ΔIVA) and ΔmPAP; **D**. correlation between ΔIVA and ΔCO in PAH patients. (delta = value during dobutamine stress test and Trendelenburg minus value at rest).

In PAH patients, in whom RV systolic function was analyzed, TAPSE correlated with mPAP and PVR (r = 0.58 and r = 0.51, respectively; *P* < 0.05), whereas, IVA and Sm were correlated with CO (r = 0.32 and r = 0.5, respectively; *P* < 0.05) (Table 5). Finally, ΔEM only correlated with ΔmPAP (r = 0.56, *P* < 0.05) and ΔIVA was correlated with ΔCO (r = 0.5, *P* < 0.05) (Figure 2).

**Table 5:** Correlations between right ventricular systolic tissue Doppler variables and hemodynamics during both conditions (rest and stress maneuver)

	**TAPSE**		**Sm**		**IVA**	
	**r**	**p Value**	**r**	**p Value**	**r**	**p Value**
mPAP	0.58	0.0004	0.24	0.09	0.17	0.72
PVR	0.51	0.002	0.26	0.08	0.19	0.16
CO	0.11	0.244	0.32	0.04	0.5	0.0026

CO: cardiac output; IVA: myocardial isovolumic acceleration; mPAP: mean and pulse arterial; PVR: pulmonary vascular resistance; Sm: myocardial peak systolic velocity; TAPSE: tricuspid annular plane systolic excursion. (r: Pearson coefficient).

The interobserver and intraobserver variabilities for IVA measurements were 4.4% and 3.4% respectively.

In the 2-year clinical follow-up there were two deaths/pulmonary transplantations in PAH group 1 and one death in PAH group 2 (*P* > 0.05).

## Discussion

This is the first study evaluating the cardiovascular reserve in PAH patients. We show that the hemodynamic response to pharmacological stress with low-dose dobutamine plus 30° Trendelenburg position is significantly impaired in patients with PAH, and this impairment is associated with a low RV systolic reserve and pulmonary vascular reserve. The lower cardiovascular reserve is significantly related to a worse hemodynamic adaptation to DST and it could be associated with a poor clinical outcome.

### Pharmacological and positional stress

Cardiovascular reserve is emerging as a strong predictor of outcome in different cardiovascular diseases [21]. From a physiological point of view, cardiovascular reserve is a measure of cardiovascular response to exercise or pharmacological stresses (dobutamine infusion between 4 and 10 mcg/kg/min) [22]. Although exercise stress is the gold standard to evaluate of the pulmonary vascular pressure-flow relationships, exercise hemodynamics in PAH patients have been poorly studied, and factors that may have an impact on PAP response to exercise, such as exercise method, exercise intensity, position and age, have not been accounted for. The stress maneuver used in our study provided by low-dose dobutamine plus 30° Trendelenburg position works by a purely passive increasing in CO without directly influence on the PA wall viscoelastic properties. In experimental pulmonary hypertension, dobutamine infusion at a rate of 10 mcg/kg/min has no flow-independent effects on the normal or acutely hypertensive circulation. Higher doses may have a constricting or dilating effect depending on the pre-existing vascular tone [22-24].

Taking into account the extent of heart rate and CO reached by healthy subjects during pharmacological and positional stress, we achieved a cardiovascular stress level similar to a slight/moderate exercise (heart rate 100–110 bpm and cardiac output 10–14 L/min) [2]. According to the data reviewed by Kovacs et al., mPAP values during slight exercise in healthy subjects were 29.4 ± 8.4 mm Hg, 20.0 ± 4.7 mm Hg and 18.2 ± 5.1 mm Hg in subjects aged ≥ 50 years, 30–50 years and less than 30 years, respectively [2]. Our healthy controls were aged 51 ± 6 years (range 40–60 years; 50% ≤ 50 years) and showed a similar mPAP (18 ± 4 mm Hg) with a similar CO increase (doubled) during DST.

### Cardiovascular reserve in PAH versus control patients

In the control group, the marked increase in CO during DST did not cause any significant change in mPAP and determined a low ΔmPAP/ΔCO ratio (0.7 ± 0.2 mm Hg/L/min). This value corresponds well to the cohort of Kovacs et al. which reported a ΔmPAP/ΔCO ~1.06 mm Hg/L/min [25].

The modest increment in mPAP relative to CO during pharmacological stress is attributable to passive recruitment and distension of a normally compliant pulmonary circulation with active flow-mediated vasodilation, decreasing PVR and TPR [15]. Studies into the regulation of pulmonary vascular tone during exercise demonstrate the importance of nitric oxide in the exercise-induced pulmonary vasodilatation, which is mediated in part via blunting of the vasoconstrictor influence of endothelin [26]. Concomitantly, pharmacological stress produced an increase in arterial pulsatility (estimated by IVUSp), and a decrease of EM, expressing a preserved buffering function. It is accepted that the arterial wall buffering function is determined not only by arterial elastic properties, but also by the viscous properties of the wall. The characterization of wall buffering function has been estimated by means of the ratio between viscous index/elastic index [27]. Considering our stress condition as a mainly passive condition (with no significant change in viscous index), a decreased EM with a negative ΔEM, would be associated with a preserved buffering function and buffering function reserve, respectively. The negative change in ΔPVR with a low ΔmPAP/ΔCO ratio reflects a preserved pulmonary vasodilator reserve. In clinical practice, ventricular systolic reserve is usually defined by a change in ejection fraction or SV during exercise or dobutamine infusion. Even though we did not assess RV function indices in the control group, the observed CO increase was composed by a 56% increase in heart rate (chronotropic reserve) and 20% (13 mL) increase in SV (systolic reserve).

Although, both controls and PAH patients had similar heart rate at rest, PAH cohort showed an impaired chronotropic response during stress maneuver. This chronotropic incompetence has been previously documented by Provencher et al. and may reflect the loss of normal physiological reserve secondary to significant autonomic nervous system abnormalities and probably as a result of down-regulation of β-adrenoreceptors [28,29].

Both PAH patient groups had similar resting hemodynamics and chronotropic reserve. However, the higher increase in mPAP and pPAP with similar increase of CO during stress in PAH group 1 with respect to PAH group 2, would be related to the reduced recruitability and distensibility of more highly remodeled pulmonary vessels. This illustrates a lack of physiological adaptation of the PA wall to increased flow in relation with a lower vasodilation reserve (positive ΔPVR), a higher PA wall remodeling (higher resting EM) and a concomitantly lower buffering function (higher ΔEM). By contrast, PAH group 2 preserved some extent of vascular reserve secondary to a vasodilation response (negative ΔPVR) and a lower impairment of buffering function (lower positive ΔEM). This would explained the significant lower ΔmPAP/ΔCO ratio than group 1. Accordingly, we have previously reported that PAH patients with higher IVUSp and lesser EM displayed an absolute PA vasodilation during acute vasoreactivity testing [18]. We cannot discard the presence of alterations in the control of pulmonary vascular tone during DST, resulting in blunted pulmonary vasodilation. Since both PAH groups have neither demographic (age, gender or body surface area) nor clinical differences (functional class, 6 minutes walking distance, etiology of PAH), we can speculate that PAH group 1 could have higher endothelial dysfunction with higher imbalance between vasodilators and vasoconstrictors than group 2, explaining the significant higher ΔmPAP/ΔCO ratio [26].

In accordance with previous data, resting hemodynamic measurements are poorly correlated with the response to pharmacological stress [30]. However, EM at rest was significantly correlated with ΔmPAP and ΔCO. Accordingly, Kubo et al. showed that the percentage of wall thickness was highly correlated with ∆mPAP during exercise in patients with severe emphysema [31]. The correlation between ΔmPAP and ΔEM (Figure 2) suggests that PA wall remodeling and buffering function impairment would be associated with the lower vascular reserve.

In the context of PAH, evidence of RV dysfunction and clinical right-sided heart failure at rest have been shown to be the most important determinants of morbidity and mortality, independently of PAP values. We used three measures of longitudinal RV shortening in an effort to characterize simple and reproducible measurements of global RV systolic function [19]. The mean reference value of TAPSE is 23 mm (16–30), Sm 15 cm ⋅ s^-1^ (10–19) and IVA 3.7 m ⋅ s^-2^ (2.2-5.2) [19]. Among them, TAPSE, a simple and clinically useful tool to estimate RV function in PAH patients, has been shown to predict survival in PAH [32]. Preliminary evidence suggests that a decrease in TAPSE with exercise was strongly associated with adverse clinical events in PAH patients within one year of follow up [33]. However, Giusca et al. suggested that tricuspid ring motion is only loosely related to RV systolic function, being highly dependent on afterload and overall motion of the heart, thus failing to reflect RV longitudinal function accurately [34]. This may explain why TAPSE changes are more significantly related to changes in mPAP and PVR than to true changes in RV systolic function such as CO. Myocardial deformation parameters provide a more accurate picture of the contractile status of the RV free wall [19,34]. IVA appears as a relatively load-independent estimator of the RV systolic response to stress, probably reflecting true changes in contractility and in CO induced by DST. Sm of RV basal free wall is also better related to CO than TAPSE. However, in this work it showed a lower ability to identify systolic reserve than IVA, since there were no Sm differences between both PAH patients groups either at rest or during stress. Although both PAH patients groups showed similar resting RV function, PAH group 1 showed a lower RV systolic reserve than PAH group 2, estimated by a lower increase in IVA and a decrease in SV during DST (P < 0.05). Systolic reserve is dependent on several factors, such as ventricular contractility, ventricular remodeling and, myocardial interstitial fibrosis. The correlation between ΔCO and ΔIVA (Figure 2) suggests that RV contractility impairment would explain a lower systolic RV reserve. However, we cannot discard a stress-induced ischemia and attenuated oxygen supply to the right myocardium during stress maneuver in more severe PAH patients that could explain their impaired RV systolic reserve [35,36].

Recently, Blumberg et al. correlated exercise hemodynamics with peak oxygen uptake and determined their prognostic significance in PAH patients. Among hemodynamic variables, only exercise cardiac index and the slope of the pulmonary pressure/flow relationship were significant prognostic indicators [11]. Therefore, the exaggerated increase in mPAP with no concomitant increase in CO (abnormal slope of the pressure/flow relationship) during DST despite similar resting hemodynamics, allows speculating a worse outcome of PAH group 1 with respect to group 2 [11].

### Study limitations

Although care must be taken when comparing hemodynamic response induced by physical exercise with pharmacological stress produced by low-dose dobutamine infusion, the similar response to exercise and to dobutamine infusion at 10 mcg/kg/min in patients with PH following the Mustard operation is compelling [37].

In addition, our pharmacological stress was a step closer to real exercise, since increased preload was achieved with the addition of 30° Trendelenburg. In fact, our stress maneuver doubled the CO in the control population. The stress with dobutamine and Trendelenburg works by a purely passive effect on PVR and PC, mimicking the CO response to moderate exercise without interfere with PA vascular tone. Invasive recordings of exercise hemodynamics in PAH require an intensive protocol, best performed by an experienced team, and thus it does not belong in the routine evaluation of PAH patients [8,24]. Our safe stressor protocol should be viewed as an easier and more reproducible maneuver than physical exercise in the catheterization laboratory.

The relative contributions of longitudinal and transverse shortening to overall RV function have been quantified recently. Although, we only assess RV systolic reserve by longitudinal shortening indices, Brown et al. showed that improved RV function following pulmonary vasodilator therapy occurs solely from improvements in longitudinal contraction, suggesting that longitudinal shortening may represent the afterload-responsive element of RV functional recovery [38]. Finally, we do not estimate a possible contribution of impaired diastolic reserve in the cardiovascular adaptation to the stress maneuver.

## Conclusions

Pulmonary vascular reserve and RV systolic reserve are impaired in PAH patients. The PA wall remodeling, pulmonary buffering function and RV contractility appeared as the main factors of the cardiovascular reserve dysfunction in PAH patients. The lower recruitable cardiovascular reserve is significantly related to a worse hemodynamic response to DST and it could be associated with a poor clinical outcome. Further study is needed to elucidate whether cardiovascular reserve dysfunction adds independent prognostic information in a multivariate analysis. In addition, further studies needs to assess whether improvement of cardiovascular reserve could be a therapeutic target in patients with established pulmonary hypertension.

## Abbreviations

CO: Cardiac output; DST: Dobutamine stress test with simultaneous 30° Trendelenburg; EM: Elastic modulus; IVA: Myocardial acceleration during isovolumic contraction; IVUS: Intravascular ultrasound; IVUSp: IVUS pulsatility; mPAP: Mean pulmonary arterial pressure; PA: Pulmonary artery; PAH: Pulmonary arterial hypertension; PC: Pulmonary capacitance; pPAP: Pulse PAP; PVR: Pulmonary vascular resistance; RV: Right ventricle; Sm: Myocardial peak velocity during ejection phase; TAPSE: Tricuspid annular plane systolic excursion; TPR: Total pulmonary resistance.

## Competing interests

The authors declare that they have no competing interests.

## Authors’ contributions

ED and JCG conceived of the study, participated in its design, conducted the study, analyze the data, and wrote the manuscript. RA participated in the design of the study, conducted the study and helped write the manuscript. CA and NB conducted the study and analyze the data. MLM conceived of the study and participated in its design. AR conceived of the study, participated in its design, and wrote the manuscript. All authors read and approved the final manuscript.

## Pre-publication history

The pre-publication history for this paper can be accessed here:

http://www.biomedcentral.com/1471-2466/14/69/prepub
